# Phenotypic diversity among juvenile polyposis syndrome patients from different ethnic background

**DOI:** 10.1186/s13053-021-00207-9

**Published:** 2022-01-20

**Authors:** Lior Haim Katz, Rachel Gingold-Belfer, Elez Vainer, Shani Hegger, Ido Laish, Estela Derazne, Ilana Weintraub, Gili Reznick-Levi, Yael Goldberg, Zohar Levi, Shlomi Cohen, Elizabeth E. Half

**Affiliations:** 1 Department of Gastroenterology and Hepatology, Hadassah-Hebrew University Medical Center, Ein Kerem, 91120 Jerusalem, Israel; 2 Faculty of Medicine, Hebrew University of Jerusalem, Jerusalem, Israel; 3 Division of Gastroenterology, Rabin Medical Center, Beilinson Hospital, Petach-Tikva, Israel; 4 Sackler Faculty of Medicine, Tel Aviv University, 6997801 Tel Aviv, Israel; 5 Department of Internal Medicine B, Rabin Medical Center, Beilinson Hospital, Petach-Tikva, Israel; 6 Department of Gastroenterology , Sheba Medical Center, Tel-Hashomer, Israel; 7 Department of Gastroenterology, RAMBAM Health Care Campus, Haifa, Israel; 8 Statistic Department, The Sackler School of Medicine, Tel-Aviv University, Tel-Aviv, Israel; 9 Division of Pediatric Gastroenterology, Hepatology and Nutrition, Edmond and Lily Safra Children’s Hospital, Sheba Medical Center, Tel-Hashomer, Israel; 10 Genetics Department, RAMBAM Health Care Campus, Haifa, Israel; 11 Genetics Department, Rabin Medical Center, Beilnson Hospital, Petach-Tikva, Israel; 12 Deparment Pediatric Gastroenterology and Nutrition Unit, The Dana Dwek Children’s Hospital, Tel-Aviv Sourasky Medical Center, Tel-Aviv, Israel

**Keywords:** Juvenile polyposis syndrome, Phenotype, Ethnic groups

## Abstract

**Abstract:**

Juvenile polyposis syndrome (JPS), has diverse phenotypes. Aim: To assess mutation rate, clinical features and genotype-phenotype correlation among Israeli JPS kindreds from different ethnicities.

**Methods:**

Patients’ data were extracted retrospectively from 5 centers.

**Results:**

Thirty five kindreds (49 patients) were included. Thirty one (89%) Jewish [10 (32%) Ashkenazi; 9 (29%) Sephardi; 11 (35%) non-Russia former Soviet-Union countries (NRFSU), one (3%) unknown]. 40/49 individuals from 27 families underwent genetic testing. Among them 34, from 21 families (85, 78%, respectively) had a pathogenic mutation: *BMPR1A n* = 15 (71%), *SMAD4 n* = 6 families (29%). While no *SMAD4* mutation was described among Jewish families from NRFSU, 7 NRFSU families carried a founder mutation comprising a large genomic deletion of *BMPR1A*. GI involvement was reported in 42 patients (86%): colonic polyps (*n* = 40, 95%, > 50 polyps *n* = 14, 35%) and 12 underwent colonic resection. Fourteen patients (34%) had gastric or small bowel involvement (*n* = 5) and 4\14 underwent gastrectomy due to polyp burden. Families from NRFSU had more gastric involvement (66.7% vs. 22.2%- Sephardic and 20%- Ashkenazi Jews; *p* = 0.038), with more gastric polyps (*p* = 0.017).

**Conclusions:**

We demonstrated a high rate of mutation detection in the heterogeneous population of Israel. Patients from NRFSU with BMPR1A mutation had high rate of gastric involvement.

## Introduction

Juvenile polyposis syndrome (JPS), first described in 1964 [1], is a rare autosomal dominant condition affecting between 1 in 100,000 and 1 in 160,000 [2–5] individuals. It is characterized by predisposition to hamartomatous polyps in the gastrointestinal (GI) tract. Most individuals with JPS have juvenile polyps by age 20 years [6] which may cause rectal bleeding and anemia. Polyps occur predominantly in the colon and rectum (98%) but can occur in the stomach (14%) and small bowel (SB) (14%) [4, 5, 7, 8]. Germline mutations in the *SMAD4* or bone morphogenetic protein receptor type-1A (*BMPR1A*) genes are identified in approximately 45–65% of JPS patients [5, 6, 9–11]. These genes are related to the transforming growth factor-beta (*TGF-β*) signaling pathway [12, 13].

Individuals with JPS are at increased risk for colorectal, gastric and small bowel cancers, which necessitate physicians and patients to adhere to lifelong surveillance with upper GI endoscopy and colonoscopy, starting at time of diagnosis [6, 8, 14]. The cumulative risk of colorectal cancer (CRC) in individuals with JPS is about 38–68% [8, 15, 16], but lower rates have been reported too [5]. CRC in JPS occurs at a younger age as compared with sporadic CRC (mean age 34 years) [16]. The incidence of gastric cancer is 21% in those with gastric polyps [6–8].

A genotype-phenotype correlation in JPS is poorly defined. While some members of the same family with the same pathogenic variant have few polyps, others may have over 100 [6]. The age of polyp development also can be extremely different among affected patients within the same family. Previous studies have shown that individuals with *SMAD4* pathogenic mutation may have an increased risk of severe gastric polyposis [9, 17] and a higher risk for gastric cancer [18]; most JPS patients with a *SMAD4* pathogenic mutations may have hereditary hemorrhagic telangiectasia (HHT) [19–21]; and people with either an *SMAD4* or *BMPR1A* pathogenic variant are more likely than those without a pathogenic variant identified to have more than ten lower GI polyps and a family history of GI cancer [6, 17, 22, 23].

Israel is known for its population diversity with people from different ethnicities and immigrants from different parts of the world share similar health care coverage. Since most of the published data on JPS is based on European and North-American studies, the aim of this study was to assess the rate of mutation, clinical features and genotype-phenotype correlation among Israeli JPS kindreds from different ethnicities.

## Methods

Individulas were identified from five adult and pediatric tertiary centers in Israel (Rambam Health Care Campus, Haifa; Dana Dwek Children’s Hospital, Tel-Aviv; Rabin Medical Center, Petach-Tikva; Sheba Medical Center, Tel-Hashomer, Ramat Gan and Hadassah Medical Center, Jerusalem) Each institution collected data for this study in accordance with a local institution-specific institutional review board (IRB) protocol. All, data was collected retrospecively from patient electronic medical records by Febuary 2019.. Included patients had JPS according to the following accepted clinical criteria [6, 24]: (1) at least five juvenile polyps in the colorectum, (2) juvenile polyps throughout the gastrointestinal tract or (3) any number of juvenile polyps in a person with a known family history of juvenile polyps, as well as patients with pathogenic mutation in *SMAD4* or *BMPR1A* from a kindred with JPS, regardless their polyp status. Genetic testing, including Sanger sequencing and multi-gene new generation sequencing panels were performed by medically certified laboratories. Surveillance protocol of all five institutes has been colonoscopy and upper GI (UGI) endoscopy every 1 to 3 years depending on polyp burden. Small bowel imaging was not part of routine surveillance. Since this study covers many years, the surveillance protocol might have been changed during the study period. Data encompassed patient demographics and family history, genotype, disease phenotype, endoscopic data, surveillance, and long-term outcomes. Polyp burden in the colon and in the stomach was grouped into five categories: 0; 1–10; 11–50; 50–99; 100 and above. Study outcomes were colonic/gastric/small bowel involvement and colonic/gastric surgery. Since only two patients underwent small bowel-associated therapeutic procedure (surgery or double balloon enteroscopy), we could not perform any statistics on this outcome.

### Statistical analysis

The characteristics of the participants and families are presented as median and range or as number and percentage for categorical variables. Separate analyses were conducted for patients and for families. For the family’s analysis we studied the participant with the most severe phenotype from families with more than one included participant. The association of study outcome with categorical variables was assessed with chi-square test (χ2) or Fisher exact test in case of 2*2 tables. The association between study outcomes and polyp number was measured by Mann-Whitney test. A *p* value < 0.05 (two-sided) was considered statistically significant. Statistical analyses were performed with IBM SPSS Statistics for Windows, version 27.0. Armonk, NY: IBM Corp.

## Results

Overall, 49 participants from 35 families were included in our study. Their baseline characteristics are described in Table 1. For 11 families more than one family member with JPS was included in the study. Among them seven families had two family members with JPS; Two had three members; and one family had four. Thirty one families (89%) were Jewish from diverse ethnicities. The others were Druze (two families), Muslim and non-Jewish Ukrainian (one family each). Among the Jewish families, 10 (32%) were Ashkenazi; 9 (29%) were Sephardi; and 11 (35%) were from non-Russia former Soviet-Union countries (NRFSU, mainly from Bukhara and Georgia). Ethnicity was not recorded in one family (3%) that was lost from follow-up. Twenty two families (63%) reported on having more than one family member with phenotypic manifestations of JPS.

**Table 1:** Baseline characteristics of the cohort

Characteristics	N (%/range)
Number of patients	49
Number of families	35
Median follow-up period (range)	5 y (< 1 to 49)
Sex – female	19 (38.8)
Median age of diagnosis (range)	13 y (2–68 y)
Ethnicity – Jewish families	31 (89)
- Ashkenazi/Sephardi/NRFSU/Unknown	10 (32)/ 9 (29)/ 11 (35)/ 1 (3)
Family history of GI cancer – families	6 (17)
Performance of genetic testing – families	27 (77)
Genetic diagnosis among tested families	21 (78)
*BMPR1A* mutation diagnosed among genetic diagnosed families	15 (71)
- “Bukharin mutation” among *BMPR1A* mutation carriers	7 (47)
*SMAD4* mutation diagnosed among genetic diagnosed families	6 (29)
- HHT symptoms among *SMAD4* families	5 (83)
Presenting symptom (symptomatic patients only) – rectal bleeding	22 (88)
Presenting symptom (symptomatic patients only) – abdominal pain	5 (20)

*NRFSU* non Russia former Soviet Union, *y* years, *HHT* hereditary hemorrhagic telangiectasia

### Genotype

**Fig. 1:**
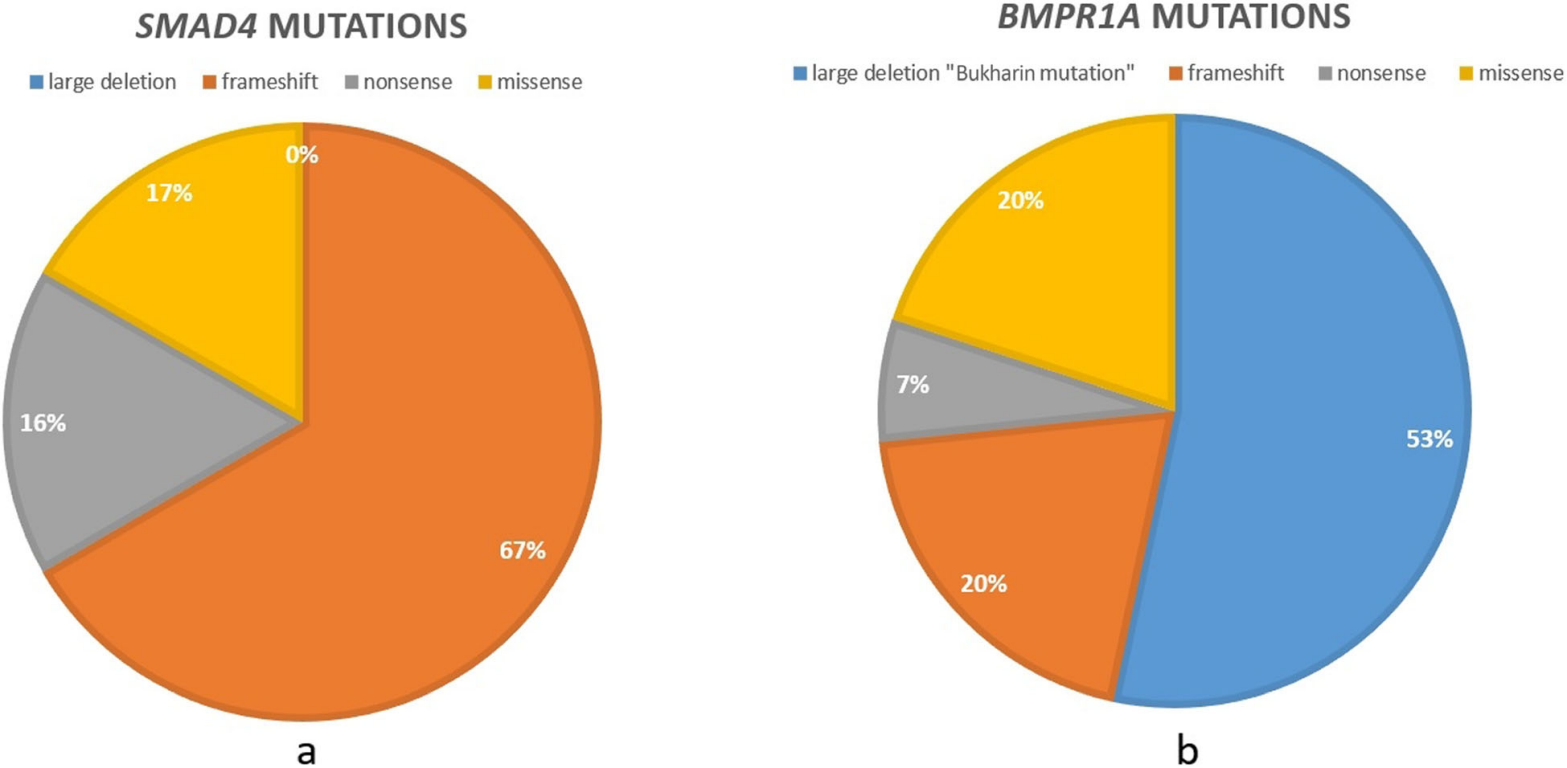
Schematic presentation of the types of mutations for each gene: (a) *SMAD4*, (b) *BMPR1A*

Forty participants (82%) from 27 families (77%) underwent genetic testing. Among the tested patients and families 34 patients from 21 families (85, 78%, respectively) were found to carry a pathogenic mutation: 15 families (71%) had a pathogenic mutation in the *BMPR1A* gene and six families (29%) in the *SMAD4* gene. (23 and 11 participants, respectively). No *SMAD4* mutation was described among Jewish families from NRFSU and among Druze families. The types of mutations for each gene are shown in Fig. 1. Among *BMPR1A* mutation carriers, seven families were from NRFSU, specifically from Bukhara (a city in Uzbekistan). The Bukharin Jewish families originate from a highly endogamous community in central Asia for some 2500 years, and immigrated to Israel after the collapse of the former Soviet Union [25]. These seven families carry a founder mutation comprising a large genomic deletion of 429,426 bp (chr10:88,611,882- 89,041,308 [hg19]), encompassing the entire coding region (exons 3–13) of *BMPR1A*, and the complete loci of 8 downstream genes [25]. Having a mutation in general, having a mutation in either gene or having a specific mutation (i.e.the *BMPR1A* Bukharin mutation) was not associated with any specific phenotype or with disease severity compared to participants who had negative genetic results (no mutation identified) or have not been tested, had a mutation in the other gene *(BMPR1A* or *SMAD4*) or had a non-*BMPR1A* Bukharin mutation, respectively.

### Clinical manifestations and phenotype

Median age of JPS diagnosis was 13 years (2–68 years). Twenty five (51%) patients were diagnosed due to JPS-related symptoms, and in 22 of them (96%) the presenting symptom was rectal bleeding. Only 5 patients (21%) reported abdominal pain, accompanied by rectal bleeding in four. In two patients iron deficiency anemia was the presenting symptoms and in another seven participants, anemia accompanied rectal bleeding. Three patients had hypoalbuminemia, and one of them had an intussusception. One child had failure to thrive and in one family genetic testing was performed as part of the evaluation of autism. HHT was diagnosed in five out of six families with *SMAD4* mutation (83%), but none of the *BMPR1A* families had HHT.

**Fig. 2:**
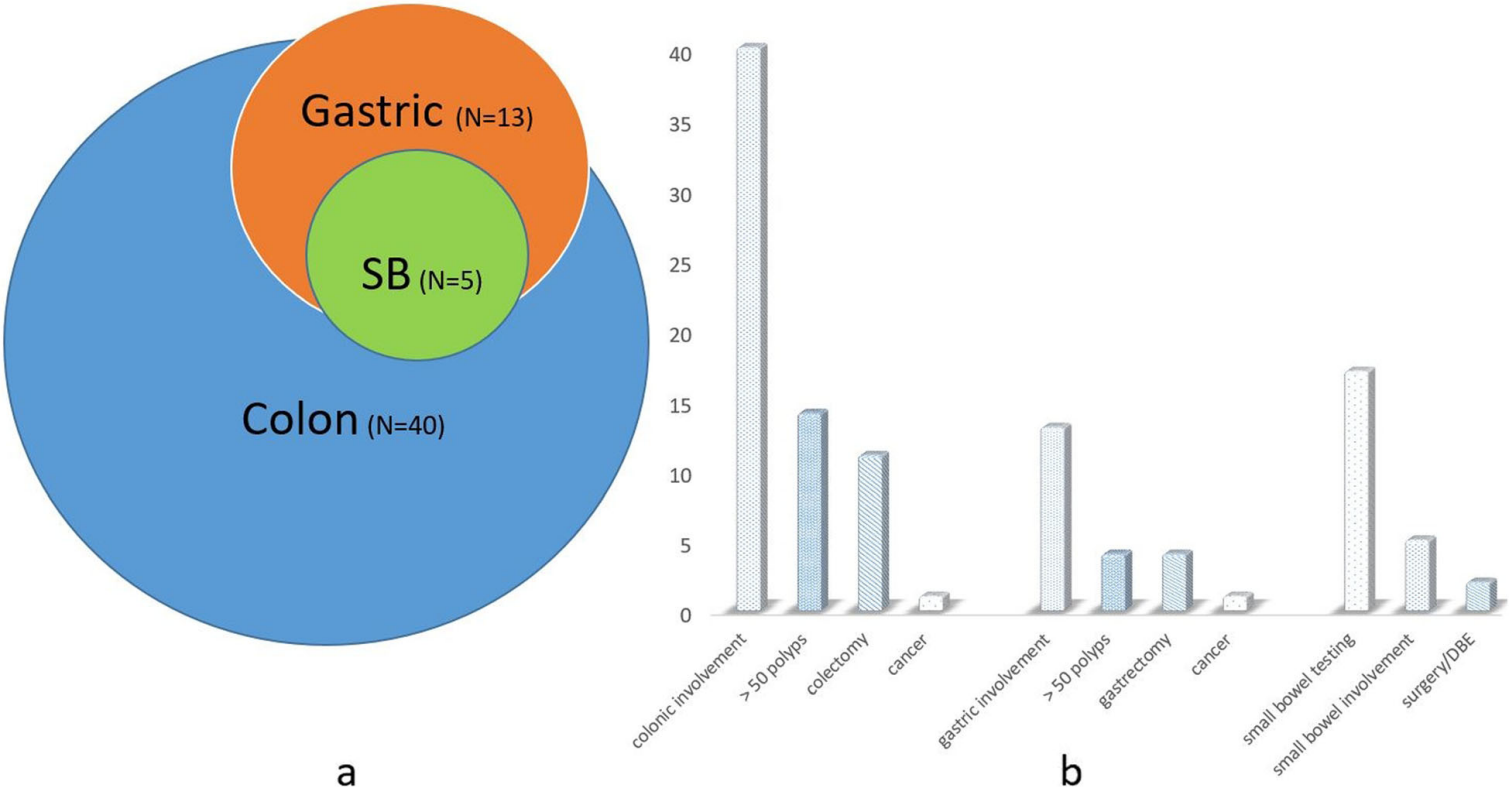
Phenotypic manifestations of Juvenile Polyposis Syndrome (a) The distribution of gastrointestinal tract involvement (b) Specific phenotype of involved organs: colon, stomach and small bowel

GI involvement was reported in 42 patients (86%): 40 with colonic polyps (95%) and 14 (33%) with upper GI involvement. Of those with upper GI involvement, 13(92%) had gastric polyps (five patients had the *BMPR1A* Bukharin mutation and four had *SMAD4* mutation). Five patients (38%) had polyps in the SB (one with the *BMPR1A* Bukharin mutation, one with *SMAD4* mutation and the other three did not undergo genetic tests or were found negative). Only two participants (15%) had gastric involvement without colonic involvement (one with the *BMPR1A* Bukharin mutation and the other one with *SMAD4*), and all five participants with SB involvement had colonic involvement as well (four of them had also gastric polyps). (Fig. 2). Hypoalbuminemia occurred only in patients with polyps in the SB (*n* = 3. Two of them with *SMAD4* mutation and one has not been tested).

Thirteen participants had between one and ten polyps; and 13 – between 11 and 50. Fourteen participants (35% of participants with colonic involvement) had more than 50 colonic polyps, and 12 of them underwent colonic resection. One patient was diagnosed with CRC at presentation: a 19 years old male with the *BMPR1A* Bukharin mutation and a high burden of colonic polyps. Colonic surgery was associated not only with polyp burden (*p* < .0001), but also with the presence of adenomas in the colon (*p* = .001) and with gastric involvement (*p* = .007) and gastric surgery (*p* = .016). A compound variable containing number of colonic polyps and the presence of adenoma was associated with colonic surgery (p = .001). Only patients with more than 10 polyps underwent colonic surgery. Of these patients 3/15 (20%) had only hamartomas while 6/9 (67%) had both hamartomas and at least one adenoma.

Four participants (31% of participants with gastric involvement) underwent gastrectomy due to a high polyp burden or cancer. Three surgical interventions were performed in non NRFSU patients and only one in an NRFSU patient. One participant was diagnosed with gastric cancer during follow-up: An Ashkenazy Jewish male, diagnosed with JPS at the age of four years and had a previous colectomy due to colonic polyps burden. The gastric cancer was diagnosed on the first UGI endoscopy, performed at the age of 32 years. Among five participants with SB involvement, it was severe enough to require surgery or polypectomy by enteroscopy in two participants (40%). Families from NRFSU had more gastric involvement (66.7% vs. 22.2% in Sephardic Jews and 20% in Ashkenazi Jews; *p* = 0.038), with more polyps in the stomach (*p* = 0.017), regardless genetic status (Table 2).

**Table 2:** The phenotypic manifestations of JPS in NRFSU families compared to Non- NRFSU families

		Non NRFSU families (%) (*N* = 23)	NRFSU families (%) (*N* = 11)	*p*-value
Genetic test performance	no	6 (26.1)	1 (9.1)	*p* = NS
	yes	17 (73.9)	10 (90.9)	
Positive genetic test	no	3 (17.6)	3 (30.0)	*p* = NS
	yes	14 (82.4)	7 (70.0)	
Affected gene	*SMAD4*	6 (42.9)	0 (.0)	*p* = NS*
	*BMPR1A*	8 (57.1)	7 (100.0)	
Colonic involvement	no	1 (4.3)	1 (9.1)	*p* = NS
	yes	22 (95.7)	10 (90.9)	
Colonic polyps	0	1 (4.3)	1 (9.1)	*p* = NS
	1–10	5 (21.7)	4 (36.4)	
	11–50	8 (34.8)	3 (27.3)	
	50–100	9 (39.1)	2 (18.2)	
	> 100	0 (.0)	1 (9.1)	
Colonic surgery	no	18 (78.3)	7 (63.6)	*p* = NS
	yes	5 (21.7)	4 (36.4)	
CRC	no	23 (100.0)	10 (90.9)	Not done
	yes	0 (.0)	1 (9.1)	
Gastric involvement	no	17 (77.3)	3 (33.3)	***p*** **= 0.038**
	yes	5 (22.7)	6 (66.7)	
Gastric polyps	0	13 (76.5)	3 (33.3)	***p*** **= 0.017**
	1–10	2 (11.8)	2 (22.2)	
	11–50	0 (.0)	2 (22.2)	
	50–100	2 (11.8)	1 (11.1)	
	> 100	0 (.0)	1 (11.1)	
Gastric cancer	no	22 (95.7)	11 (100.0)	Not done
	yes	1 (4.3)	0 (.0)	
Gastric surgery	no	20 (87.0)	10 (90.9)	*p* = NS
	yes	3 (13.0)	1 (9.1)	
Small bowel involvement	no	8 (80.0)	2 (40.0)	*p* = NS
	yes	2 (20.0)	3 (60.0)	
Surgery/DBE/SBE	no	14 (87.5)	6 (100.0)	*p* = NS
	yes	2 (12.5)	0 (.0)	
Family history of cancer	no	10 (83.3)	3 (42.9)	*p* = NS
	yes	2 (16.7)	4 (57.1)	

*NRFSU* non Russia former Soviet Union, *NS* not significant, *CRC* colorectal cancer, *DBE* double balloon enteroscopy, *SBE* single balloon enteroscopy

*SMAD4 mutation showed a trend towards negative association with *p* = 0.057

## Discussion

JPS is a relatively rare polyposis syndrome. Here we describe genotypic and phenotypic analysis of 49 JPS patients from 35 families in Israel. Israel’s population is composed of multinational immigrants which makes it a very diverse population. It includes Jewish people from different ethnicities as well as non-Jewish population. We show a high representation of patients from NRFSU, most of them sharing a founder mutation. These patients tend to have higher rates of gastric involvement. We report a higher rate of positive genetic tests (78%) among tested families, compared to older publications [9–11]. Only a minority of our patients (10%) had SB involvement, all of them had colonic polyps as well.

According to older literature the rate of positive genetic tests is between 45 and 65% [5, 6, 9–11]. Our results demonstrate higher rates, similar to those described in St. Marks series (14/17 kindreds, 82%) [5]. These higher rates can be attributed to better quality of the genetic tests as well as to including MLPA or other methods for detecting large deletions as a part of the genetic tests. In adition we cannot rullout a selection bias due to the fact that all thses indicvidulas were refferd from tertiary referal centers. However due to the social medicalsystem in Israel where gentic testing for JPS is available free of charge to all individulas who answere clinical criteria this is highly unlikely. … .

While no patient with *SMAD4* mutation in our study had a large deletion, seven kindreds (46.7%) had a large deletion in the *BMPR1A* gene. All besides one family had the *BMPR1A* Bukharin mutation. According to a recent publication from Europe [26] the rate of large deletions in the *SMAD4* is reported to be higher in *SMAD4* and lower in *BMPR1A* (6.7–21.4% large deletions in SMAD4 and 13.5–16.4% in *BMPR1A*). This reflects the specific ethnicity background in Israel which is different from that in Europe.

As expected, we did not find any difference between *BMPR1A* and *SMAD4* carriers in terms of colonic phenotype and polyp burden. Surprisingly, in contrast to previous series which showed higher gastric polyp rate and more severe gastric phenotype among *SMAD4* mutation carriers [9, 17, 26], we did not find such an association. Apparently, the reason for this finding is the dominance of the *BMPR1A* Bukharin mutation among our *BMPR1A* mutation carriers. As previously reported [24] this mutation does not carry a risk for more severe phenotype, but is characterized by gastric involvement which is different that other mutations in BMPR1. Indeed, we found gastric polyps in 4/7 (57%) families with the *BMPR1A* Bukharin mutation, and only in 6/28 (21%) other families. Furthermore, gastric involvement was more common in NRFSU Jewish patients compared to all other ethnicities (6/9 [66.7%] vs. 5/22 [23%] *p* = 0.038), regardless the *BMPR1A* Bukharin mutation, while none of the patients with *SMAD4* mutation was from NRFSU origin.

Overall, 17 patients had SB evaluation by CTE, MRE or capsule endoscopy, and five (10%) had SB polyps. All of them had colonic polyps as well. In two recent publications the rate of SB involvement was even lower (4.5–5.7%) [26, 27]; however older data showed 14% of JPS patients to have SB polyps [4, 5, 7, 8, 27]. Although we did not have any case of SB cancer, SB cancer has been previously described [27]. According to ACG guidelines [8] the small bowel beyond the ligament of Treitz should be periodically surveilled, depending on initial polyp findings, by enteroscopy, capsule endoscopy, and/or CT enterography if duodenal polyposis is present or if there is unexplained anemia, protein-losing enteropathy, or other SB symptoms. Additional data from larger studies is needed to establish the extent of SB involvement in JPS and the association between colonic and SB involvement.

Overall, 15 colonic and gastric surgeries were undertaken in 13 patients (26.5%). The association between colonic surgery and number of polyps is trivial; however, we also found that colonic surgery is associated with the presence of adenomas in the colon. These two variables may be correlated; however, due to small number of cases we were unable to perform a multivariate analysis. The association between colonic surgeries and gastric involvement and surgeries may indicate a severe phenotype of the syndrome. We did not find any association between this severe phenotype, patients’ origin or mutated gene. Close follow-up by both colonoscopy and UGI endoscopy is recommended for patients with the severe phenotype.

Two cases of cancer were detected in our cohort (4%) while in previous studies higher rates between 9 and 50% are reported [5, 12, 15, 26]. The reason for our lower rate of cancer diagnosis in our cohort is most probably due to the relatively higher representation of young patients (median age of JPS diagnosis in our cohort was 13 years with 5 years follow up, while in the two cohorts that were recently published the median age was 25 and 27 years, respectively [5, 26]). The median age of cancer diagnosis was 41–47 years in previous studies [5, 15, 26]; however, only 12 patients from our cohort (24%) have reached this age range. Another study from Israel described only one JPS patient with cancer (2.8%). This was a SB adenocarcinoma in a 65 years-old male [27]. The lower cancer rate in the two Israeli studies can potentially be a consequence of environmental factors, genetic modifiers or a meticulous and active surveillance program with timely polypectomies to prevent cancer development.

Our study is the first to comprehensively describe the genotypic and phenotypic manifestations of JPS in a population composed mostly of families that did not originate in European or North American ancestry. Therefore, we present data on gastric involvement in *BMPR1A* kindreds, different from what is known, including previous publications based on extensive literature search [26]. Our study included five large tertiary centers representing the Israeli population, and data were collected from both adult and pediatric GI units. Since it is based on diagnoses from recent years the genetic tests performed were more robust than those described in older studies and the mutation rate was higher than previously described.

Our study has some limitations. First, small sample size which precluded us from performing more intense statistical analyses. Nevertheless, this is a rare genetic syndrome with limited information available in the literature and we believe it represents the Israeli population since the data was taken from five large centers from different geographical locations in Israel representing the diverse country population. Among data available from Europe, included in a recent publication [26], only Germany reported a larger cohort.

Another limitation is the retrospective design of the study. This design reflects real-world data, in which not all included patients underwent UGI endoscopy and SB investigation; however, no prospective cohort of JPS kindreds has been published to date. Ascertainment and selection bias may occur more frequently in retrospective studies, as well as mishandling of data records including incomplete or missing data for some of the patients. In order to decrease the missing rate we had minimal data requirements that included ethnicity, genotype and all colonic and gastric phenotypic data.

In summary, in a retrospective cohort of 49 patients from 35 different families we have shown that in Israeli kindreds with JPS the rate of positive mutation in either *SMAD4* or *BMPR1A* is high, approaching 80%. There is high representation of kindreds from NRFSU with a unique phenotype that includes gastric involvement in *BMPR1A* mutation carriers. Larger studies are needed to measure the actual mutation rate in JPS kindreds in 2021 and to assess the genotype-phenotype association in JPS families from Israel as well as from other non-European cohorts.

## Data Availability

The datasets used and analysed during the current study are available from the corresponding author on reasonable request.
